# Collaborative Control for a Robot Manipulator via Interaction-Force-Based Impedance Method and Extremum Seeking Optimization

**DOI:** 10.3390/s25247648

**Published:** 2025-12-17

**Authors:** Ming Pi

**Affiliations:** School of Information and Control Engineering, Southwest University of Science and Technology, Mianyang 621010, China; pm198709@mail.ustc.edu.cn; Tel.: +86-15280986919; Fax: +86-0816-6089312

**Keywords:** robot manipulator, adaptive impedance control, extremum seeking method

## Abstract

This paper introduces an adaptive impedance control strategy for robotic manipulators, developed through the extremum seeking technique. A model-based disturbance observer (DOB) is employed to estimate contact forces, removing the dependency on torque sensors. An impedance vector is constructed to correct the errors arising from motor uncertainties and unknown couplings, without considering the threshold value of the control parameters. Joint tracking errors and fluctuations in contact force are incorporated into the cost function. For various tasks, suitable control parameters are adaptively optimized in real time using an extremum seeking approach, which continuously evaluates the cost function. A rigorous analysis is conducted on the stability of the proposed controller. Compared to conventional approaches, the proposed adaptive impedance control offers a more streamlined design for adjusting the manipulator’s contact impedance. Experimental results confirm that the extremum seeking strategy successfully tuned the controller parameters online according to variations in the cost function.

## 1. Introduction

Robot manipulators are primarily designed to aid humans in carrying out tasks in intricate industrial processes, such as assembly and drilling [1,2], which typically involve environmental contact. Controlling the contact force is therefore key to ensuring rapid and precise tracking performance [3,4]. However, the presence of unmodeled disturbances and system uncertainties can hinder the robot’s ability to follow desired trajectories accurately when influenced by environmental forces.

Hence, some robust methods were proposed to avoid inaccurate and unstable motions of the manipulator, such as dynamic coupling switching [5], state space [6], and trajectory optimization [7]. Moreover, an adaptive robust control method was developed to address environmental uncertainties in the manipulator’s contact force regulation [8]. The impedance control method, serving as an alternative for manipulator contact force regulation, has also been presented [9,10]. When a robot contacts its environment, the interaction was typically modeled as a mass–spring–damper system [11].

To tackle the aforementioned issues, researchers have introduced adaptive control strategies for accurate robot impedance regulation, such as sliding-mode control (SMC) [12], time-delay control (TDC) [13], and neural network controls [14]. In [15], a distributed model-free adaptive control scheme was employed to investigate the uncertain dynamics of systems subjected to deception attacks. By leveraging the contraction mapping principle, the boundedness of the uncertain dynamics under external disturbances was rigorously demonstrated without relying on global topological information. However, the parameters of the control method were set in advance and many tests were required to achieve good control performance. In [16], an adaptive triggering mechanism with time-varying thresholds was proposed. Corresponding control protocols were designed to operate without global information, therefore ensuring that the control strategy remains effective. However, the variation in the adaptive law was fixed and could not be changed for different working conditions. In [17], the leaderless consensus problem of system with uncertain dynamics was studied. Group model learning was accomplished for Euler–Lagrange systems operating over directed networks, even under persistent unknown disturbances. However, the limits of unknown disturbances should be given in advance. In [18], a model reference adaptive consensus strategy was proposed and relied solely on position information, with the relative positions being exchanged. A drawback, however, was that the adaptive law’s parameters were fixed and require extensive testing.

SMC was more robust against the system’s nonlinear uncertainties and unknown disturbances [19]. If the SMC switching gain was larger than the highest level of nonlinear uncertainty, accurate motion regulation could be ensured. Nevertheless, it was usually impossible to define such bounds precisely [20]. When the switching gains largely exceed the upper bound, severe chattering can occur. Consequently, the robot vibrates around the sliding manifold, leading to energy loss and reduced tracking performance. A time-delay estimation (TDE) method was developed to mitigate the effects of coupled dynamics and unmodeled disturbances. Time-delay control (TDC) was a simple control scheme that had been extensively applied in autopilot systems [21] and robot control [22]. Nevertheless, TDE errors still existed, which degraded the control performance. To address this issue, appropriate control gains of the TDC were selected [23]. If the gains were excessively large or too small, the controller’s effectiveness was weakened. To decrease the effort to achieve suitable gains, neural networks were adaptively utilized to address dynamic uncertainties [24]. A supervisory fuzzy sliding mode controller was introduced for surgical robots [25]. This supervisory fuzzy control enabled real-time dynamic adjustment of the controller gains, thereby minimizing the time needed for tuning. Within a predefined range of control gains, the TDE error was successfully eliminated.

To adaptively optimize robotic motion performance, a cost function was commonly formulated for adjusting control parameters. In [26], the continues phase variable was used to regulate the impedance performance across walking modes, with a metabolic cost-based function tuning joint parameters—though updates occurred slowly. In [27], a data-driven method was conducted for stance phase with the kinematic control for swing phase. The parameters were updated to achieve the minor cost function. In [28], a novel impedance controller was designed to optimize system energy consumption. While, the optimization process required several hours. In [29], a human-in-the-loop approach was adopted to identify personalized assistance strategies, yet experiments revealed that a metabolic rate-based cost function was unsuitable for real-time adaptation. In [30], a composite cost function incorporating tracking error and energy consumption was proposed to evaluate contact force variations. Nevertheless, this function suffered from significant updated delays, highlighting the need for efficient real-time optimization methods. In [31], an extremum seeking method was employed to minimize the cost function and adjust control parameters for achieving desired robotic motion. However, the cost function relied solely on tracking error, limiting its adaptability to diverse working conditions. In [32], an extremum seeking strategy enabled real-time parameter tuning without prior knowledge or expert intervention, optimizing the cost function by adjusting manipulator stiffness in real time. Nonetheless, it neglected the influence of force variations on stiffness regulation. In [33], extremum seeking was applied to optimize a composite metric of muscular activity and spring stiffness, enabling autonomous adjustment of joint stiffness across different gait conditions. However, the control parameters responded too slowly to dynamic environmental changes. In [34], extremum seeking was utilized to adaptively adjust the control law’s parameters deduced from functional neuromuscular electrical stimulation. Although it achieved online optimization of the cost function equilibrium, the function design remained relatively simplistic, primarily based on tracking error variations.

Central Pattern Generators (CPGs), identified in both invertebrate and vertebrate species, offered an effective mechanism for producing rhythmic locomotion patterns [35,36]. Their notable features comprised self-sustained rhythmicity, bounded cycle convergence, and smooth modulation of output trajectories [37]. Typically implemented through oscillator networks, CPGs provided a tractable framework for generating and synchronizing joint trajectories. Furthermore, a CPG-based approach has been developed to estimate gait cycle percentage and derive virtual knee angles via interpolation from a healthy knee’s motion profile [38].

This paper established an adaptive impedance control scheme for robot manipulators. An impedance vector is introduced to formulate a simplified control structure. The traditional adaptive impedance control strategies usually only consider tracking the desired contact force and do not take into account the suppression of joint tracking errors. This paper proposed a comprehensive cost function *J* that considers both the tracking of desired contact forces and the suppression of joint tracking errors, and an extremum seeking strategy is employed to dynamically optimize the manipulator’s control parameters in response to cost function changes.

The key contributions of this study are summarized as follows: (1) the design of a model-based disturbance observer (DOB) for contact force estimation; (2) the formulation of a cost function integrating joint tracking errors and contact force variations; (3) the application of an extremum seeking approach to optimize control parameters by minimizing the cost function; (4) the adaptive control framework’s performance in manipulator motion tasks was verified experimentally.

## 2. Materials and Methods

### 2.1. Dynamic Model of Robot Manipulator

**Figure 1:**
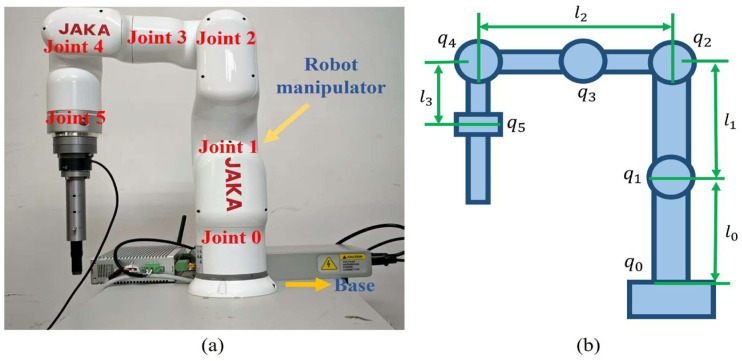
Sketch of the robot manipulator. (**a**) Real robot manipulator. (**b**) Diagram of manipulator’s parameters.

The 4-DOF robotic manipulator incorporates two active joints. As illustrated in Figure 1, the rotation angles of joints 1 and 2 are denoted as *q*_1_ and *q*_2_, respectively. The joint-space dynamics of the manipulator are expressed as follows:

(1) $$M\ddot{q}+C\dot{q}+G=\tau +\varpi_{e}$$

where $q={\left[q_{1},q_{2}\right]}^{T}\in {\mathbb{R}}^{2}$ represents the rotation angle of the robot joint. $\tau \in {\mathbb{R}}^{2}$ denotes the control signal of the robot joint. $\varpi_{e}\in {\mathbb{R}}^{2}$ denotes the interaction torque between the level ground and robot. $M\in {\mathbb{R}}^{2\times 2}$ denotes the inertia matrix, $M=\left[\begin{array}{cc} m_{1}{d_{1}}^{2}+I_{1}+I_{2}+P_{11} & m_{2}{d_{2}}^{2}+I_{2}+P_{12}\\ m_{2}{d_{2}}^{2}+I_{2}+P_{21} & m_{2}{d_{2}}^{2}+P_{22} \end{array}\right]$, $P_{11}=m_{2}({l_{1}}^{2}+{d_{2}}^{2}+2l_{1}l_{2}\cos q_{2})$, $P_{12}=m_{2}l_{2}d_{2}\cos q_{2}$, $P_{21}=m_{2}\;l_{2}\;d_{2}\;\cos q_{2}$, $P_{22}=I_{2}$; $C\in {\mathbb{R}}^{2\times 2}$ denotes the centripetal-Coriolis matrix, $C=\left[\begin{array}{cc} -m_{2}l_{1}d_{2}\sin q_{2}{\dot{q}}_{2} & -m_{2}l_{1}d_{2}\sin q_{2}({\dot{q}}_{1}+{\dot{q}}_{2})\\ m_{2}l_{1}d_{2}\sin q_{2}{\dot{q}}_{1} & 0 \end{array}\right]$; $G\in {\mathbb{R}}^{2}$ is the gravitational torque, $G=\left[\begin{array}{c} Q_{1}+l_{1}\cos q_{1}\\ Q_{2} \end{array}\right]$, $Q_{1}=m_{1}d_{1}g\cos q_{1}+m_{2}d_{2}g\cos(q_{1}+q_{2})$, $Q_{2}=m_{2}d_{2}g\cos(q_{1}+q_{2})$.

The parameters of robotic manipulator can be designed as: $m_{1}$ = 3.5 kg, representing the mass of link 1; $m_{2}$ = 3.5 kg, representing the mass of link 2; $d_{1}$ = 0.25 m, connecting the center to joint 1; $d_{2}$ = 0.25 m, connecting the center to joint 2; $I_{1}$ = 1.3 kg·m^2^, representing the inertia of link 1; $I_{2}$ = 1.9 kg·m^2^, representing the inertia of link 2; $l_{1}=0.52$ m, connecting joint 1 to joint 2; $l_{2}=0.52$ m, connecting joint 2 to the end-effector; and g=9.8 m/s^2^, representing gravitational acceleration. The primary variable notations are summarized in Table 1.

**Figure 2:**
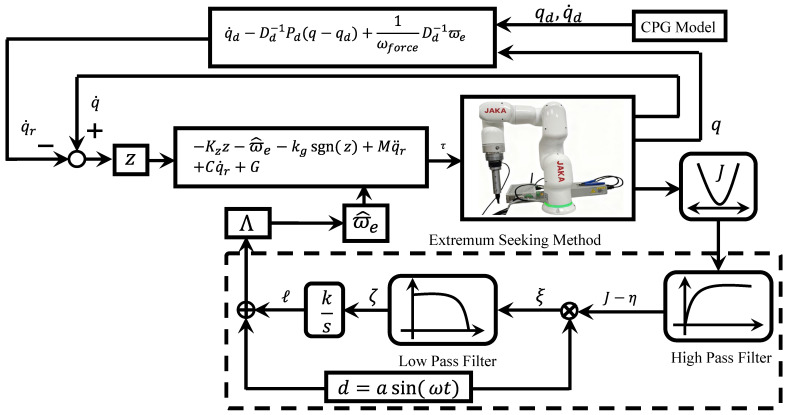
Schematic representation of the control flow. The reference trajectory $q_{r}$ denotes the input trajectory and synthesized based on $q_{d}$ and $\tau^{e}$. q denotes the output trajectory. Through optimization, $q_{r}$ is adaptively adjusted to reduce the cost function J. Parameter estimation enables the controller to maintain accurate tracking.

This method aims to adaptively adjust the robot’s joint trajectory $q_{d}$ to achieve a balance among tracking errors, contact force variations, and changes in $\varpi_{e}$. The tuning process for robot manipulator motion is illustrated in Figure 2. Hence, the CPG model was employed to generate joint trajectories for various motion tasks. The joint trajectory can be immediately regulated by changing the state values of the CPG model.

According to [39], the functional mechanism of the CPG model can be summarized as:

(2) $$q_{di}=r_{0}+\sum_{j=1}^{N}r_{j}\sin(\omega_{j}t+s_{j})$$

(3) $${\dot{\omega }}_{j}=g_{j}\left[\frac{g_{j}}{4}\left(\Omega_{j}-\omega_{j}\right)-\omega_{j}\right]$$

(4) $${\dot{s}}_{j}=g_{j}\left[\frac{g_{j}}{4}(S_{j}-s_{j})-s_{j}\right]$$

(5) $${\dot{r}}_{j}=g_{j}\left[\frac{g_{j}}{4}(R_{j}-r_{j})-r_{j}\right]$$

(6) $${\dot{r}}_{0}=g_{0}\left[\frac{g_{0}}{4}(R_{0}-r_{0})-r_{0}\right]$$

where $q_{di}$ representing the desired trajectory of the manipulator’s i-th joint, is generated by superimposing N oscillators. $r_{0}$ represents the total oscillator offset. N is the total number of oscillators. j serves as the oscillator’s index. The variables $r_{j}$, $\omega_{j}$, and $s_{j}$ denote the amplitude, frequency, and phase of j-th oscillator, respectively. Constants $g_{j}$ and $g_{0}$ set the convergence speed. Furthermore, $\Omega_{j}$, $R_{j}$ and $S_{j}$ represent the sine function of $\omega_{j}$, $r_{j}$, $s_{j}$, respectively; $R_{0}$ signifies its total offset. According to the Fourier series expansion, we have

(7) $$\Omega_{j}=j\omega_{1}$$

(8) $$S_{j}=\arctan\frac{a_{j}}{b_{j}}$$

(9) $$R_{j}=\sqrt{a_{j}^{2}+b_{j}^{2}}$$

(10) $$R_{0}=a_{0}$$

(11) $$a_{j}=\frac{2}{T}\int_{0}^{T}q_{i0}\cos jw_{1}tdt$$

(12) $$b_{j}=\frac{2}{T}\int_{0}^{T}q_{i0}\cos jw_{1}tdt$$

(13) $$a_{0}=\frac{2}{T}\int_{0}^{T}q_{i0}dt$$

where $w_{1}=\frac{2\pi }{T}$, T represents the motion cycle, $q_{i0}$ represents the initial trajectory. $\Omega_{j}$, $R_{j}$, and $S_{j}$ can be calculated, $\omega_{j}$, $r_{j}$, and $s_{j}$ will converge to $\Omega_{j}$, $R_{j}$ and $S_{j}$ respectively [39].

### 2.2. Extremum Seeking Optimization

The composite cost function integrates trajectory tracking errors and contact force variations, formulated as follows:

(14) $$J=\frac{\lambda }{T}\overset{m}{\underset{i}{\sum }}{\left(q_{di}-q_{i}\right)}^{2}+\frac{1-\lambda }{T}(\overset{m}{\underset{k}{\sum }}s_{k}^{2}+\alpha \overset{m}{\underset{i}{\sum }}\varpi_{e,i}^{2})$$

where i, m, and k represent the index of time point, $s_{k}$ denotes the strength of the contact force at the k-th time point, α denotes a positive gain. The initial term guarantees that the manipulator follows the generated trajectory, and the second term aids in lowering the manipulator’s interaction torque with the environment. The weight between the two terms is predefined by the constant gain λ∈(0, 1). When λ=0, the emphasis of optimization is on trajectory alignment. When λ=1, the emphasis transfers to optimizing the contact force $\varpi_{e}$.

An extremum seeking strategy is introduced to optimize the composite cost function J. The selected matrices and their associated costs are collated into $W_{past}$ and $J_{past}$ respectively. Minimizing (14) yields a variable and individualized trajectory to help the manipulator match the correct and appropriate tracking trajectory. The initial weight matrix was specified based on historical data. The extremum seeking method is illustrated in Figure 2. A sinusoidal probing signal, formulated as d(t)=asin(ωt), was employed to estimate the critical parameter l. According to (18) and (19), l serves as the critical variable for estimating ${\hat{\varpi }}_{e}$. l governs the behavior of the motor control at the joint. Subsequently, the cost function *J*, derived from Equation (14), undergoes signal processing to extract the key parameter l. The main process for the extremum seeking optimization is formulated as follows: first, the resulting signal J−η is calculated; secondly, $\dot{\eta }$, $\dot{\xi }$, and $\dot{l}$ are updated according to $\dot{\eta }=\omega_{h}(J-\eta )$, $\dot{\xi }=-\omega_{l}\xi +\omega_{l}(J-\eta )a\sin(\omega t)$, and $\dot{l}=k\xi$, $\omega_{1}$ and $\omega_{h}$ denote positive gains, $l=l_{pre}+\dot{l}$, $\xi =\xi_{pre}+\dot{\xi }$, and $\eta =\eta_{pre}+\dot{\eta }$, $l_{pre}$, $\xi_{pre}$ and $\eta_{pre}$ represent pre-estimated values of each corresponding variable; then, Λ is calculated from Λ=lI; finally, ${\hat{\varpi }}_{e}$ can be regulated.

The control architecture was illustrated in Figure 2, consisting of three primary functional modules: (1) the reference trajectory $q_{r}$ denotes the input trajectory and synthesized based on $q_{d}$ and $\tau_{e}$; (2) through optimization, $q_{r}$ is adaptively adjusted to reduce the cost function J; (3) parameter estimation enables the controller to maintain accurate tracking.

### 2.3. Control Development

#### 2.3.1. Interaction-Force-Based Impedance Method

We assume that $s_{k}$ denotes the strength of k-th time point of the contact force. The weight function is designed as follows:

(15) $$\omega_{force}=c_{1}\tan h(-\frac{\sum_{k}^{m}s_{k}^{2}}{\iota_{1}}+\iota_{2})+c_{2}$$

where $c_{1}$ and $c_{2}$ denote the positive gains to regulate the range and median of $\omega_{force}$, respectively. $\iota_{1}$ and $\iota_{2}$ denote the positive gains to regulate the range and median of $\sum_{k}^{m}s_{k}^{2}$.

Subsequently, the impedance model based on the contact force can be expressed as:

(16) $$D_{d}(\dot{q}-{\dot{q}}_{d})+P_{d}(q-q_{d})=\frac{1}{\omega_{force}}\varpi_{e}$$

where $D_{d}$ and $P_{d}$ represent the damping and stiffness matrices, respectively. (16) permits the robot joint to deviate from the reference trajectory $q_{d}$ and adjust the tracking trajectory accordingly. When the change in the contact force increases, $\omega_{force}$ is reduced, thus lowering the impedance parameters and resulting in a large deviation.

Hence, we designed an impedance vector based on the interaction torque $\varpi_{e}$ as:

(17) $$z=\dot{q}-{\dot{q}}_{r}=\dot{q}-{\dot{q}}_{d}+D_{d}^{-1}P_{d}\left(q-q_{d}\right)-\frac{1}{\omega_{force}}{D_{d}}^{-1}\varpi_{e}$$

where ${\dot{q}}_{r}={\dot{q}}_{d}-D_{d}^{-1}P_{d}(q-q_{d})+\frac{1}{\omega_{force}}D_{d}^{-1}\varpi_{e}$ denotes the designed value. According to (17), when z→0, (16) achieves the reference tracking performance.

Moreover, to avoid the use of a force sensor to measure $\varpi_{e}$, the estimation $\varpi_{e}$ was established by a model-based disturbance observer (DOB):

(18) $$\left\{\begin{array}{l} \dot{y}=-B(\dot{q},q)y-B(\dot{q},q)\left[-C\dot{q}-g+p\right]\\ {\hat{\varpi }}_{e}=y+p \end{array}\right.$$

where ${\hat{\varpi }}_{e}$ denotes the estimated $\varpi_{e}$, y and p denote the designed values, and $B(\dot{q},q)$ denotes the gain matrix. Gain matrix $B(\dot{q},q)$ and vector p can be formulated as follows:

(19) $$\left\{\begin{array}{l} B(\dot{q},q)=\Lambda^{-1}M^{-1}\\ p=\Lambda^{-1}\dot{q} \end{array}\right.$$

where Λ denotes an invertible gain matrix, Λ=lI, l denotes the positive gain to be determined by $l=l_{pre}+\dot{l}$. We assumed ${\tilde{\varpi }}_{e}$ to be the observation error, that is ${\tilde{\varpi }}_{e}={\hat{\varpi }}_{e}-\varpi$. The adaptive law for ${\hat{\varpi }}_{e}$ can be expressed as:

(20) $$\dot{\tilde{\varpi }}=-B(\dot{q},q)\tilde{\varpi }-{\dot{\varpi }}_{e}$$

The overall control input is designed as:

(21) $$\tau =-K_{z}z-{\hat{\varpi }}_{e}-K_{g}\cdot \mathrm{sgn}(z)+M{\ddot{q}}_{r}+C{\dot{q}}_{r}+G$$

where $k_{g}$ denotes the positive constant gain, and $K_{z}$ ∈ ${\mathbb{R}}^{\mathrm{nxn}}$ denotes the positive constant matrix. sgn(⋅) denotes the sign function, $\mathrm{sgn}(z)=\left\{\begin{array}{l} 1,\;\;\;\;\;z>0\\ 0,\;\;\;\;z=0\\ -1,\;\;z<0 \end{array}\right.$. By substituting (17) and (21) into (1), we obtain:

(22) $$M\dot{z}+Cz=-K_{z}z-{\tilde{\varpi }}_{e}-k_{g}\mathrm{sgn}(z)$$

#### 2.3.2. Stability Analysis

To guarantee the stability of the control system, the overall system should be bounded, and the quasi-steady-state system should be exponentially stable. The stability of the control system can be analyzed using the selected Lyapunov function as follows:

(23) $$\gamma_{1}=\frac{1}{2}z^{T}Mz$$

Combining (22) and (23), the following equation can be obtained:

(24) $${\dot{\gamma }}_{1}=-z^{T}K_{z}z-z^{T}{\tilde{\varpi }}_{e}-k_{g}z^{T}\mathrm{sgn}(z)$$

when $\left\|{\dot{\varpi }}_{e}\right\|\leq \varsigma$, the observation error is also bounded, and:

(25) $${\dot{\gamma }}_{1}\leq -z^{T}K_{z}z-(k_{g}-\kappa )\|z\|$$

(26) $$\kappa \geq \frac{2\varsigma \lambda_{\max}(M){\left\|\Lambda \right\|}^{2}}{\rho \lambda_{\min}(\Gamma )}$$

where κ denotes the limitation of the uncertainty $\frac{2\varsigma \lambda_{\max}(M){\left\|\Lambda \right\|}^{2}}{\rho \lambda_{\min}(\Gamma )}$, $k_{g}$ denotes a positive value. When $k_{g}>\kappa$, we have

(27) $${\dot{\gamma }}_{1}\leq -z^{T}K_{z}z<0$$

when ${\dot{\gamma }}_{1}<0$, the system was exponentially stable.

Moreover, we consider the following candidate Lyapunov function.

(28) $$\gamma =\gamma_{1}+{\tilde{\varpi }}_{e}^{T}\Lambda^{T}M\Lambda {\tilde{\varpi }}_{e}$$

Combining (28) and (20), the following equation can be obtained:

(29) $$\dot{\gamma }={\dot{\gamma }}_{1}-{\tilde{\varpi }}_{e}^{T}(\Lambda +\Lambda^{T}-\Lambda^{T}\dot{M}\Lambda ){\tilde{\varpi }}_{e}+{\dot{\varpi }}_{e}^{T}\Lambda^{T}M\Lambda {\tilde{\varpi }}_{e}+{\tilde{\varpi }}_{e}^{T}\Lambda^{T}M\Lambda {\dot{\varpi }}_{e}$$

Considering the bounds of $\left\|{\dot{\varpi }}_{e}\right\|$, $\left\|{\dot{\varpi }}_{e}\right\|<\gamma$, γ denotes the bound. (29) can be formulated as follows:

(30) $$\begin{array}{l} \dot{\gamma }\leq {\dot{\gamma }}_{1}-\lambda_{\min}(\Gamma ){\left\|\tilde{\varpi }\right\|}^{2}+2\gamma \lambda_{\max}(M){\left\|\Lambda \right\|}^{2}\left\|{\tilde{\varpi }}_{e}\right\|\\ \;\;={\dot{\gamma }}_{1}-(1-\hbar )\;\lambda_{\min}(\Gamma ){\left\|{\tilde{\varpi }}_{e}\right\|}^{2}-\hbar \lambda_{\min}(\Gamma ){\left\|{\tilde{\varpi }}_{e}\right\|}^{2}\\ \;\;+2\gamma \lambda_{\max}(M){\left\|\Lambda \right\|}^{2}\left\|{\tilde{\varpi }}_{e}\right\| \end{array}$$

where ℏ∈(0, 1), $\Gamma =\Lambda +\Lambda^{T}-\Lambda^{T}\dot{M}\Lambda$. Because $\gamma -\gamma_{1}={\tilde{\varpi }}_{e}^{T}\Lambda^{T}M\Lambda {\tilde{\varpi }}_{e}\leq$$\;\lambda_{\max}(M){\left\|\Lambda \right\|}^{2}{\left\|{\tilde{\varpi }}_{e}\right\|}^{2}$, when $\left\|{\tilde{\varpi }}_{e}\right\|\geq \frac{2\gamma \lambda_{\max}(M){\left\|\Lambda \right\|}^{2}}{\hbar \lambda_{\min}(\Gamma )}$, (30) can be deduced as:

(31) $$\dot{\gamma }\leq {\dot{\gamma }}_{1}-(1-\hbar )\lambda_{\min}(\Gamma ){\left\|{\tilde{\varpi }}_{e}\right\|}^{2}\leq {\dot{\gamma }}_{1}-\frac{\left(1-\hbar )\lambda_{\min}(\Gamma \right)}{\lambda_{\max}(M){\left\|\Lambda \right\|}^{2}}(\gamma -\gamma_{1})$$

Then, we have:

(32) $$\dot{\gamma }-{\dot{\gamma }}_{1}\leq -\frac{\left(1-\hbar )\lambda_{\min}(\Gamma \right)}{\lambda_{\max}(M){\left\|\Lambda \right\|}^{2}}(\gamma -\gamma_{1})$$

And, the exponential convergence can be formulated as:

(33) $$\gamma (t+\Delta t)-\gamma_{1}(t+\Delta t)\leq (\gamma (t)-\gamma_{1}(t))\exp(-\frac{\left(1-\hbar )\lambda_{\min}(\Gamma \right)}{\lambda_{\max}(M){\left\|\Lambda \right\|}^{2}}\Delta t)$$

where $\Delta \gamma =\gamma -\gamma_{1}$ achieved exponential convergence. According to (27), $\gamma_{1}$ achieves exponential convergence and $\gamma =\gamma_{1}+\Delta \gamma$ also achieves exponential convergence.

## 3. Results

### 3.1. Experiments Setup

**Figure 3:**
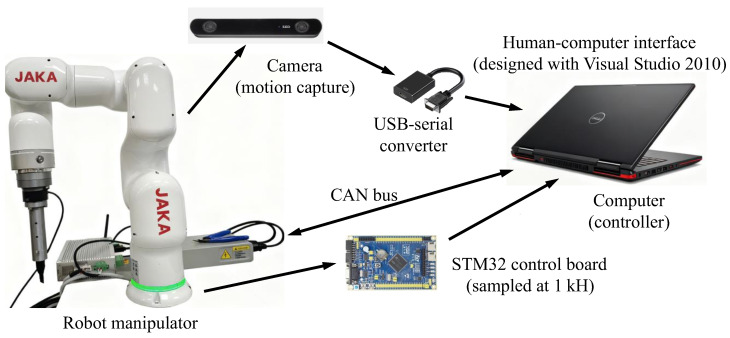
Experimental setup.

To evaluate the performance of the developed controller, three distinct motion tasks were implemented. An actual experimental setup is depicted in Figure 3. A lightweight collaborative robot manipulator was used to conduct the experiments at the SWUST Robotic Laboratory. The robotic manipulator comprises three core subsystems: a mechanically engineered framework, an integrated control unit, and a sensory feedback system. The mechanical framework was 3D printed. Table 2 provides the component masses, with a total manipulator mass of 10.8 kg. The proposed control method was validated using two active joints of the 6-DoF robotic manipulator (JAKA Robot Limit Corporation, Chengdu city, China). The first active joint operates within an approximate range of −90° to 90°, while the second joint spans roughly −150° to 150°. A DC motor actuates each joint, with an encoder measuring rotational angle data. The force sensor evaluates the change in the contact force.

### 3.2. Case 1: Manipulator Tracking a Circular Motion

(1) Experiment: The circular motion tracking task in Case 1 control strategy. A circular path was defined as the reference trajectory, which the robot’s end-effector was commanded to track. However, owing to the influence of contact force, the proposed impedance method regulated the desired trajectory to the reference trajectory. Finally, the controller computed motor torques to drive the end-effector along the reference trajectory while maintaining a consistent contact force level. The combined cost function J was designed with tracking errors and the contact force change. An extremum seeking approach is introduced to optimize the composite cost function J. The suitable control parameters are selected to help the manipulator match the correct and appropriate tracking trajectory.

**Figure 4:**
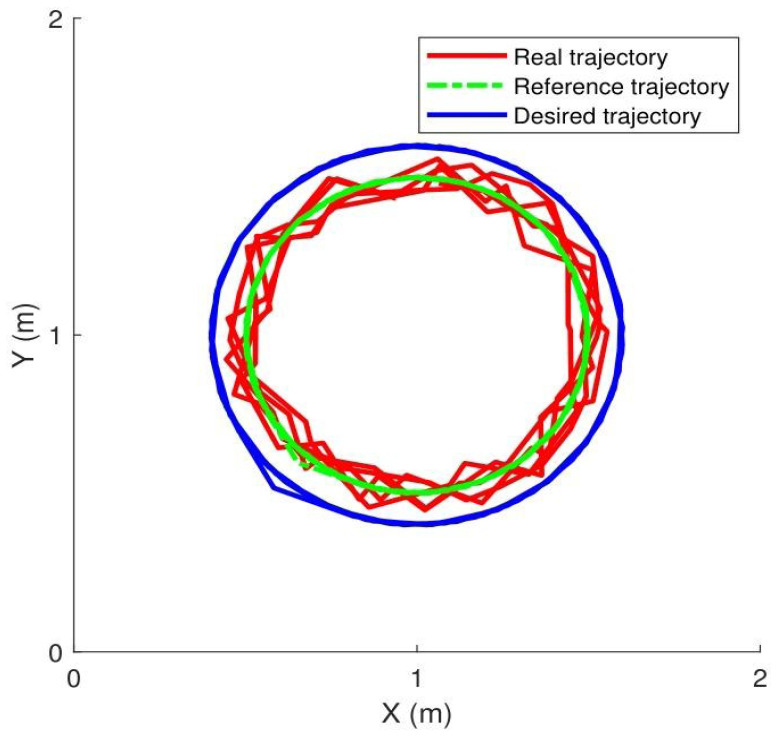
Desired circular path trajectory.

Figure 4 shows the manipulator tracking the reference trajectory, while the contact force was maintained at 50 N, 100 N, and 150 N. The main control parameters were set as: $K_{z}=3.2$, $K_{g}=1.1$, $K_{d}=\left[\begin{array}{cc} 1.3 & 0\\ 0 & 1.3 \end{array}\right]$, $C_{d}=\left[\begin{array}{cc} 0.75 & 0\\ 0 & 0.75 \end{array}\right]$.

**Figure 5:**
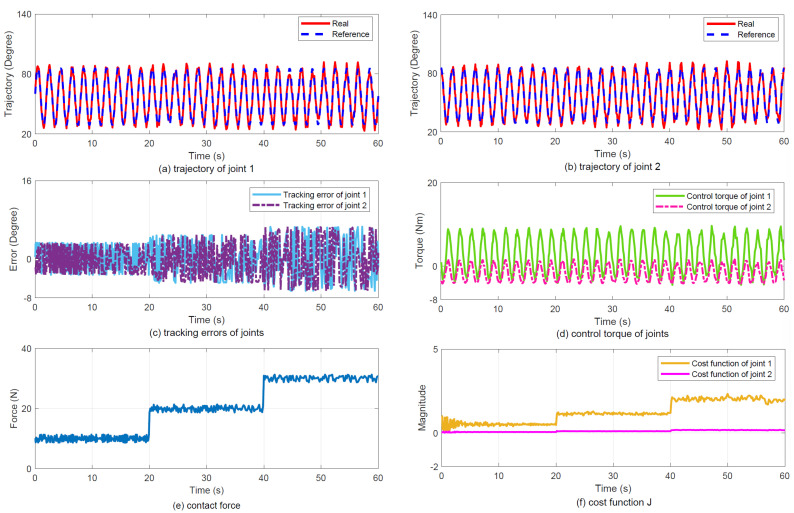
Tracking performance under Case 1. (**a**) Joint 1 trajectory tracking. (**b**) Joint 2 trajectory tracking. (**c**) Joint tracking errors. (**d**) Joint control torque. (**e**) Contact force response. (**f**) Cost function J variation.

(2) Results: Figure 5 illustrates the experimental tracking performance, with subplots (a) and (b) displaying joint trajectories, (c) presenting joint tracking errors, (d) showing control torque profiles, (e) depicting contact force variations, and (f) demonstrating the evolution of cost function J. The results indicate that the proposed controller achieves smoothly varying force regulation.

Table 3 provides a comparative summary of tracking performance for multiple controllers. The proposed control method with extremum seeking significantly outperformed torque- and voltage-based controllers, reducing the mean error (MEAN) and mean square error (MSE) to 2.7° and 1.52°, respectively, indicating enhanced tracking accuracy and stability.

### 3.3. Case 2: Manipulator Tracking a Triangular Motion

(1) Experiment: Case 2 evaluates how the proposed controller performs in a triangular circular path tracking task. The desired trajectory was designed as a triangular circle. The robotic end-effector was regulated to follow the predefined trajectory. However, due to the effect of contact force, the proposed impedance method aligned the desired trajectory with the reference trajectory. The controller computed the motor torques necessary to guide the robotic end-effector along the reference trajectory. In the process, the manipulator maintained the contact force at a fixed level. The combined cost function J was designed with tracking errors and the contact force change. An extremum seeking strategy is employed to optimize the aggregate cost function J. The suitable control parameters are selected to help the manipulator match the correct and appropriate tracking trajectory.

**Figure 6:**
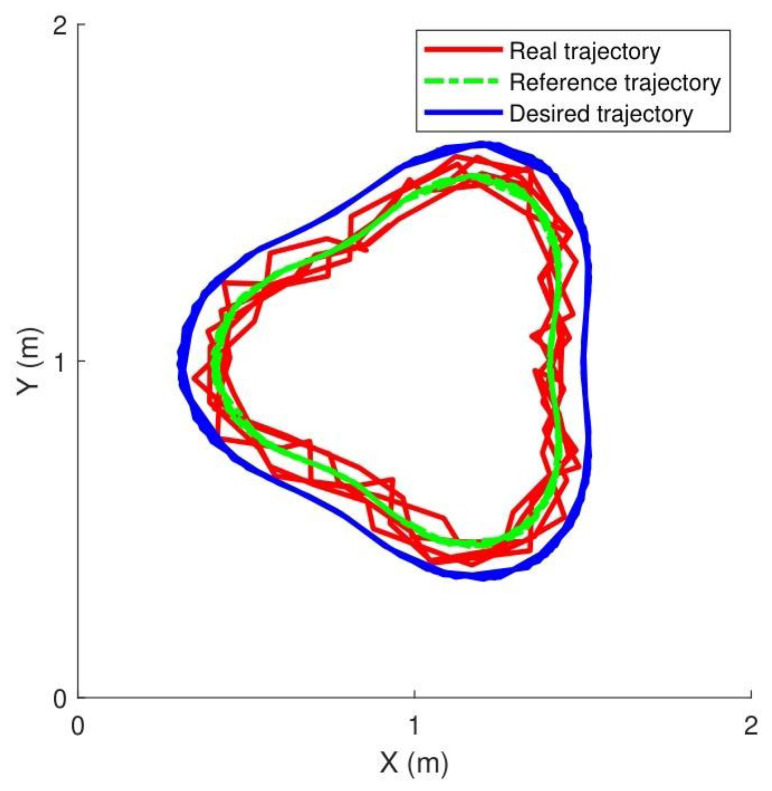
Desired triangular path trajectory.

Figure 6 shows the manipulator executing its motion by tracking the reference trajectory, while the contact force was controlled at 50 N, 100 N, and 150 N. The main control parameters were set as: $K_{z}=3.15$, $k_{g}=1.3$, $K_{d}=\left[\begin{array}{cc} 1.3 & 0\\ 0 & 1.3 \end{array}\right]$, $C_{d}=\left[\begin{array}{cc} 0.75 & 0\\ 0 & 0.75 \end{array}\right]$.

**Figure 7:**
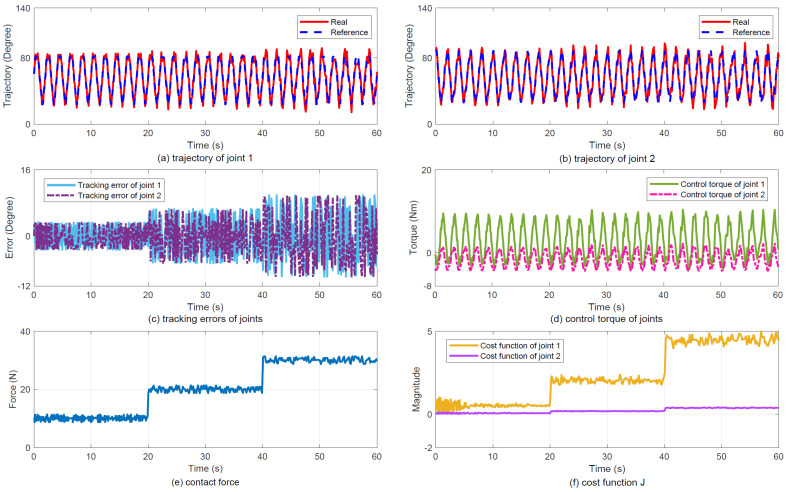
Tracking performance in Case 2. (**a**) Joint 1 trajectory tracking. (**b**) Joint 2 trajectory tracking. (**c**) Joint tracking errors. (**d**) Joint control torque. (**e**) Contact force response. (**f**) Cost function J variation.

(2) Results: Figure 7 summarizes the experimental tracking performance, with subfigures (a) and (b) illustrating joint tracking results, (c) displaying joint errors, (d) presenting control torque, (e) showing contact force variations, and (f) depicting the evolution of cost function J. The results demonstrate smooth force regulation achieved by the proposed controller.

Table 4 provides a comparative summary of tracking performance for multiple controllers. The proposed control method with extremum seeking outperformed torque-based and voltage-based approaches, lowering the mean error (MEAN) to 3.4° and the mean square error (MSE) to 1.6°.

### 3.4. Case 3: Manipulator Tracking a Sine Circular Motion

(1) Experiment: Case 3 evaluates the controller’s performance through a sinusoidal circular path tracking task, where the robot’s end-effector is guided along a predefined sinusoidal trajectory. However, due to the effect of contact force, the proposed impedance method regulated the desired trajectory to the reference trajectory. The controller computed the motor torques required to drive the robotic end-effector along the reference trajectory. In the process, the contact force was maintained at a fixed level. The combined cost function J was designed with tracking errors and the contact force change. An extremum seeking strategy is introduced to optimize the aggregate cost function J. The suitable control parameters are selected to help the manipulator match the correct and appropriate tracking trajectory.

**Figure 8:**
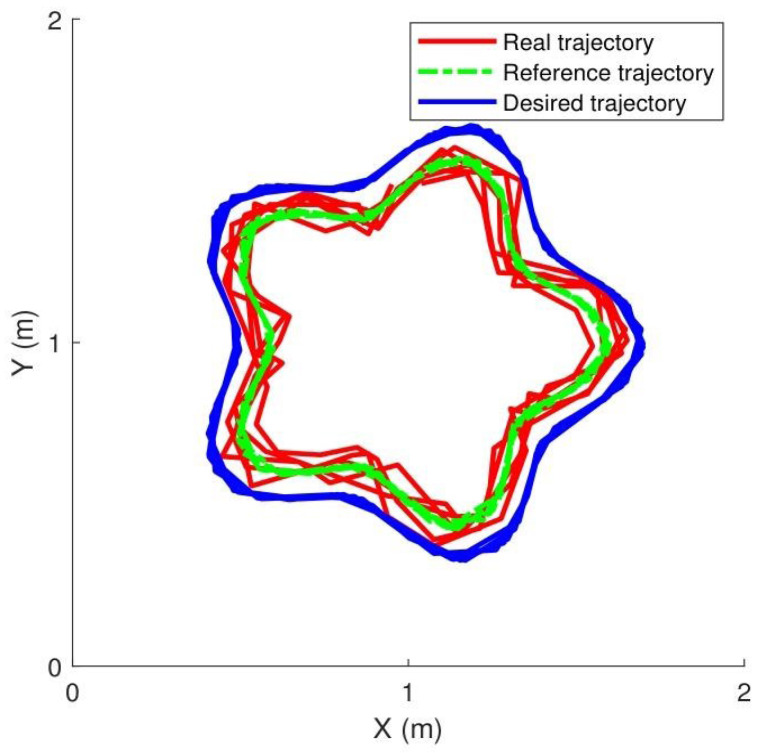
Desired sinusoidal path trajectory.

Figure 8 depicts the manipulator following the reference trajectory during its motion, while the contact force was set to 50 N, 100 N, and 150 N. The main control parameters were set as: $K_{z}=3.2$, $k_{g}=1.2$, $K_{d}=\left[\begin{array}{cc} 1.1 & 0\\ 0 & 1.1 \end{array}\right]$, $C_{d}=\left[\begin{array}{cc} 0.72 & 0\\ 0 & 0.72 \end{array}\right]$.

**Figure 9:**
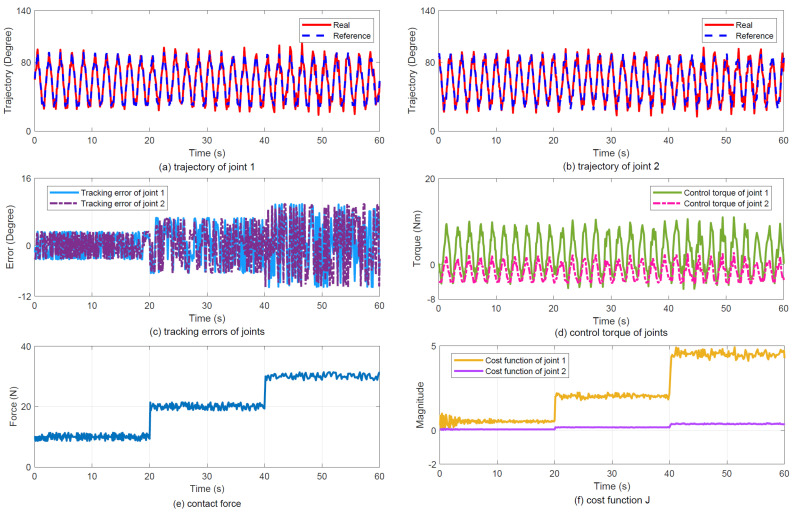
Tracking performance in Case 3. (**a**) Joint 1 trajectory tracking. (**b**) Joint 2 trajectory tracking. (**c**) Joint tracking errors. (**d**) Joint control torque. (**e**) Contact force response. (**f**) Cost function J variation.

(2) Results: Figure 9 presents the experimental tracking performance, with subfigures (a) and (b) illustrating joint trajectories, (c) displaying tracking errors, (d) showing control torque profiles, (e) depicting contact force variations, and (f) demonstrating the evolution of cost function J. The results confirm smooth force regulation achieved by the proposed controller.

Table 5 provides a comparative summary of tracking performance for multiple controllers. The proposed control method with extremum seeking outperformed torque- and voltage-based methods, lowering the mean error (MEAN) to 3.65° and the mean square error (MSE) to 2.32°.

## 4. Discussion

A model-based disturbance observer was designed to estimate the contact force and use the adaptive estimates as input terms in the tracking controller. The input–output data was exploited to achieve estimates. Some delay may be occurred at the begin of the estimation.

The tracking performance of traditional adaptive control relies on the threshold value of control parameters. This threshold must be set sufficiently large to ensure accurate tracking, but it may induce undesirable chattering. To address this issue, an adaptive control law for the threshold is designed to eliminate chattering. In comparison to conventional approaches, the developed adaptive impedance control strategy achieves a more streamlined framework for adjusting the manipulator’s impedance during environmental interaction. By integrating a model-based disturbance observer and an extremum seeking method to compensate for dynamic uncertainties, this approach achieves more robust performance.

## 5. Conclusions

This study presents an adaptive extremum-seeking control scheme for manipulator impedance regulation. The proposed impedance vector compensates for motor uncertainties and unknown couplings. A cost function was formulated to jointly account for joint tracking errors and variations in contact forces. For adaptation to different tasks, the extremum seeking method continuously modified the control parameters based on real-time variations in the cost function. Stability analysis confirmed that the control scheme achieved uniform ultimate boundedness in a semi-global sense. Experimental validation further showed that the proposed approach adaptively tuned the manipulator’s parameters, forming a natural adaptive policy. In performance tests, the mean error (MEAN) and mean square error (MSE) decreased to 2.7° and 1.52° (Case 1), 3.4° and 1.6° (Case 2), 3.65° and 2.32° (Case 3), respectively.

Future work includes enhancing the robustness of the developed controller to more external disturbances that can affect the convergence of the adaptive estimates. In addition, the designed controller will be implemented with different patterns to examine the convergence of the estimates of the parameters and assess the performance for tracking diverse tasks.

## Figures and Tables

**Table 1:** Main variable notations.

Variable	Notation	Variable	Notation	Variable	Notation	Variable	Notation
M	inertia matrix	$q_{1}$	angle for joint 1	$R_{j}$	amplitude	$l_{1}$	positive gain
C	centripetal coriolis matrix	$q_{2}$	angle for joint 2	$g_{0}$	constant	$l_{2}$	positive gain
G	gravitational torque	g	gravity	$R_{0}$	total offset	$c_{1}$	positive gain
q	real angle of joints	$q_{d}$	desired trajectory	$q_{i0}$	raw trajectory	$c_{2}$	positive gain
τ	control torque	$q_{di}$	the i-th joint of $q_{d}$	T	gait cycle	$\gamma_{1}$	candidate function
$\varpi_{e}$	interaction torque	$r_{0}$	offset	J	cost function	γ	candidate function
$q_{r}$	reference trajectory	$r_{j}$	amplitude	λ	weight	Δγ	candidate function
$P_{d}$	stiffness matric	$\omega_{j}$	frequency	Γ	assistant variable	l	control gain
$D_{d}$	damping matric	t	time	$K_{z}$	constant matrix	ζ	control gain
$\psi_{m\;s,\;0}$	initial value	$s_{j}$	phase	$k_{g}$	constant gain	η	control gain
z	impedance vector	$g_{j}$	constant	B	gain matrix	Λ	gain matrix
d(t)	seeking signal	$\Omega_{j}$	frequency	${\hat{\tau }}_{e}$	estimation		

**Table 2:** Mass weight of robot manipulator.

Part	Mass (Kg)	Motion Range (°)
body structure	2.06	
control assembly	2.38	
joint 1	2.77	−90° to 90°
joint 2	2.59	−150° to 150°
total	10.8	

**Table 3:** Trajectory tracking in case 1.

Controllers	Tracking Error (°) MEAN (°)	MSE (°)	Force Error (N) MEAN (N)	MSE (N)
Torque-based Control [10]	4.2	3.1	3.4	2.3
Voltage-based control [40]	3.6	2.3	3.0	2.5
Proposed control	2.7	1.52	2.3	1.6

**Table 4:** Trajectory tracking in case 2.

Controllers	Tracking Error (°) MEAN (°)	MSE (°)	Force Error (N) MEAN (N)	MSE (N)
Torque-based Control [10]	5.1	3.3	3.4	2.5
Voltage-based control [40]	4.6	2.5	3.4	2.35
Proposed control	3.4	1.6	2.4	2.1

**Table 5:** Trajectory tracking in case 3.

Controllers	Tracking Error (°) MEAN (°)	MSE (°)	Force Error (N) MEAN (N)	MSE (N)
Torque-based Control [10]	4.87	3.17	3.83	2.4
Voltage-based control [40]	4.3	3.2	3.6	2.3
Proposed control	3.65	2.32	2.8	1.95

## Data Availability

Data are contained within the article.
